# Primary Neuroendocrine Carcinoma of the Breast: Histopathological Criteria, Prognostic Factors, and Review of the Literature

**DOI:** 10.1155/2016/6762085

**Published:** 2016-10-20

**Authors:** Lena Marinova, Doroteya Malinova, Snezhinka Vicheva

**Affiliations:** ^1^Department of Radiotherapy, Oncology Center, Varna, Bulgaria; ^2^Department of General and Clinical Pathology, Forensic Science and Deontology, Medical University, Varna, Bulgaria; ^3^Department of General and Clinical Pathology, Oncology Center, Varna, Bulgaria

## Abstract

We present here a case of a 42-year-old woman diagnosed with primary neuroendocrine carcinoma of the breast (NECB). We discuss the importance of histological criteria for primary neuroendocrine mammary carcinoma, established by WHO in 2003 and 2012. After an overview of different cases of primary neuroendocrine carcinoma of the breast published in the literature, we present information about differential diagnosis, prognostic factors, and surgical and adjuvant treatment. Prognosis of NECB is not different from that of other invasive breast carcinomas and the most important prognostic factor is tumor grade (G). There is no standard treatment and patients should be treated similarly to patients with invasive ductal carcinoma, NOS (not otherwise specified), whose choice of therapy depends on tumor's size, degree of differentiation, clinical stage, and hormonal status.

## 1. Introduction

Primary neuroendocrine carcinomas of the breast (NECB) are rare, with incidence under 0.1% from all breast carcinomas and under 1% from all neuroendocrine carcinomas [1–6]. Focal neuroendocrine differentiation could be observed in various histologic subtypes of mammary carcinoma, including* in situ* carcinoma and invasive ductal, lobular, colloid, or papillary carcinoma [7]. According to WHO, neuroendocrine carcinoma is a tumor with positive immunoreactivity to neuroendocrine markers in at least 50% of tumor cells [8]. In 2012, WHO classified these tumors into three categories: (1) well differentiated neuroendocrine tumor; (2) poorly differentiated/small cell carcinoma; (3) invasive breast carcinoma with neuroendocrine differentiation [9]. Presence of a ductal* in situ* component is a histological evidence that breast is the primary organ of origin [3]. The average age of onset is 64 years [2, 10]. Sapino et al. describe 5 histological subtypes of NECB: solid, alveolar, small cell, solid papillary, and mucinous [11]; some of these variants were also described by other authors [10, 12]. Clinical studies on primary NECB are a few and are mostly retrospective or present individual case reports [13, 14].

## 2. Case Presentation

**Figure 1:**
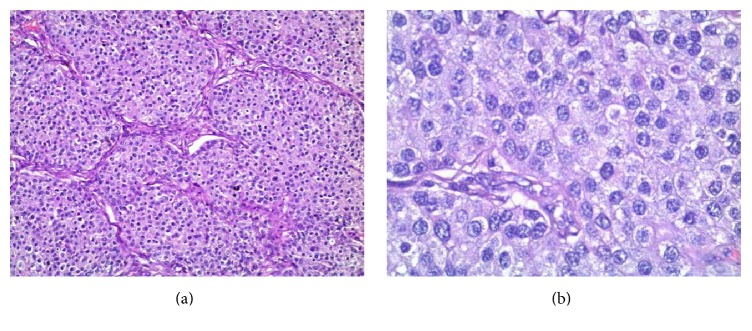
Lymph node with metastasis: (a) uniform cells, growing in solid nests, with eosinophilic cytoplasm and stippled chromatin Н&Е, ×40; (b) “salt and pepper” chromatin Н&Е, ×100.

**Figure 2:**
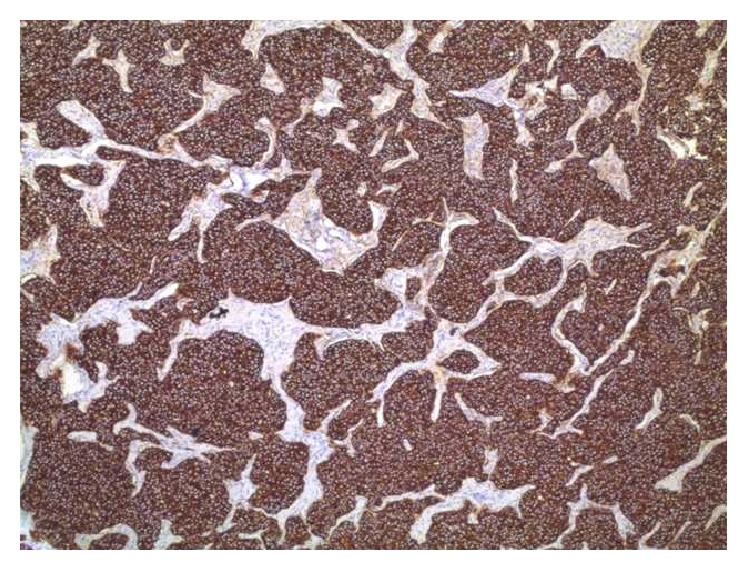
Lymph node with metastasis, nests of tumor cells, 100% expression of synaptophysin, ×40.

**Figure 3:**
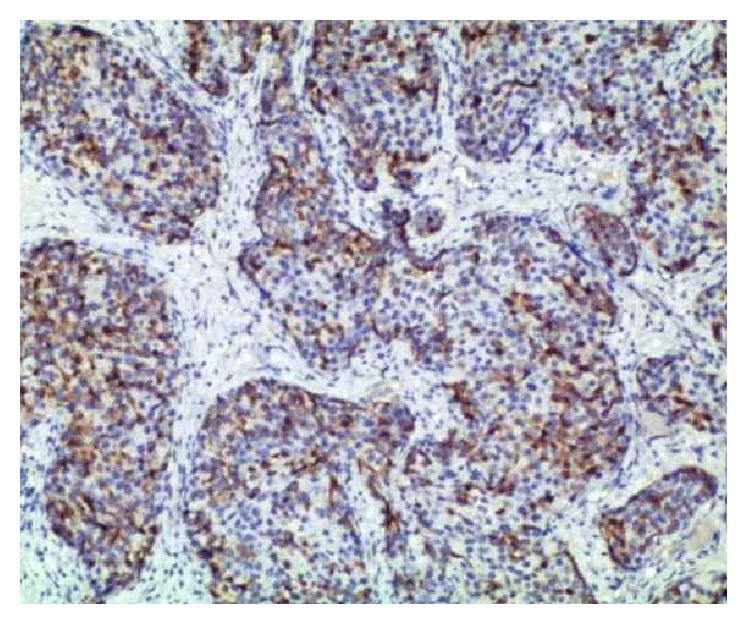
Lymph node with metastasis, nests of tumor cells, 50% expression of chromogranin А, ×40.

We present here a case of a 42-year-old woman initially diagnosed with lipoma in the right axilla. However, during the operation, three enlarged and suspicious lymph nodes were removed. Their histological examination revealed metastasis from neuroendocrine carcinoma with unknown primary site. Morphological findings included the following: solid nests of uniform tumor cells with “salt and pepper” chromatin (Figure 1). Tumor cells were positive for synaptophysin (reactivity rate 100%) (Figure 2) and chromogranin A (reactivity rate 50%) (Figure 3). After immunohistochemical analysis, the initial diagnosis was metastasis from primary pulmonary neuroendocrine tumor (large cell variant). TTF1 marker was not expressed by the tumor cells; however, it is positive in only 50% of primary lung neuroendocrine tumors.

**Figure 4:**
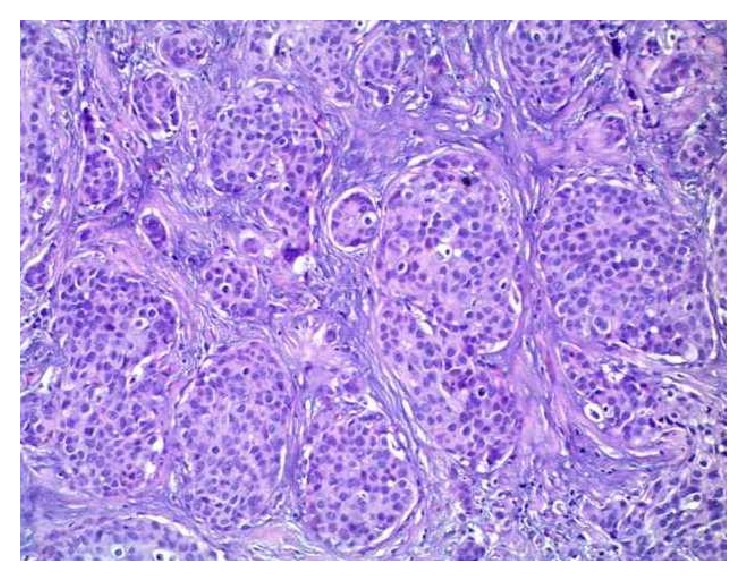
Primary tumor in breast, solid nests of uniform tumor cells with “salt and pepper” chromatin, Н&Е, ×40.

**Figure 5:**
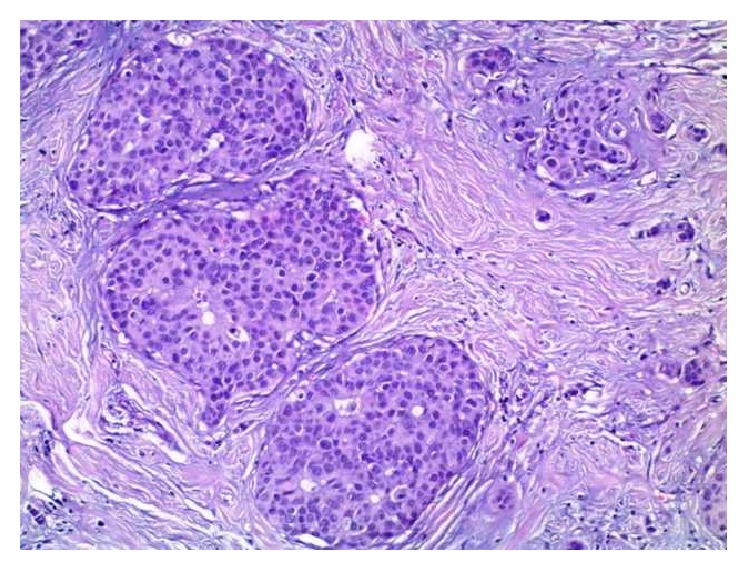
Primary tumor in breast,* in situ* component of the tumor with solid and cribriform pattern, Н&Е, ×40.

Mammography was crucial for the final diagnosis. It revealed a distinctive mass with microcalcifications in the right mammary gland measuring 35/20/10 mm. CT scan, abdominal ultrasound, and PET/CT excluded a nonmammary primary site. The patient underwent a right radical mastectomy with axillary lymph-node dissection. Histologically, the resected tumor was characterized by large uniform cells, growing in solid nests, with eosinophilic cytoplasm and stippled chromatin (Figure 4).* In situ* component of the tumor was found, which is important for the diagnosis (Figure 5). Tumor cells were positive for estrogen and progesterone receptors: HER-2 IHC test: 2+; HER-2 CISH test: negative. Biopsy examination also revealed tumor emboli in lymph vessels. Final diagnosis was solid primary neuroendocrine carcinoma of the right breast, pT2N2Mх G3. The patient received adjuvant chemotherapy (Epirubicin, Endoxan, and Fluorouracil), radiotherapy, and hormonal therapy. At one-year imaging and clinical follow-up, patient had no evidence of metastasis.

## 3. Discussion

The WHO estimates that NECB incidence varies between 0.3% and 0.5% [15, 16]. There are over 80 patients with NECB reported in the literature [12]. These tumors are thought to arise from endocrine differentiation of breast carcinoma rather than from preexisting endocrine cells with malignant transformation [12]. In 2003, WHO classified neuroendocrine neoplasms into four categories: small cell carcinoma, large cell carcinoma, solid neuroendocrine tumor, and atypical carcinoid [17]. In 2012, WHO submitted three categories: well differentiated neuroendocrine tumor, poorly differentiated/small cell carcinoma, and invasive breast carcinoma with neuroendocrine differentiation. According to the definition, NECB is a tumor expressing neuroendocrine (NE) markers in more than 50% of the cell population, synaptophysin and/or chromogranin [4–6, 8, 17, 18]. This definition includes NEBC variants which may coexpress mucinous and/or apocrine phenotype [18]. Diagnosis requires two more criteria: metastatic neuroendocrine carcinoma must be ruled out clinically and demonstration of* in situ* component histologically [19]. The breast* in situ* component is an intraductal lesion, dilated ducts with the luminal spaces completely filled with ovoid, spindle-shaped, or polygonal cells with low- or moderate-grade atypia [20].

Histologically neuroendocrine tumors are characterized by uniform cells (round- or spindle-shaped), nuclear palisading, abundant finely granular eosinophilic cytoplasm, and nuclei with “salt and pepper” chromatin. Tumor cells form nests, islands, and alveolar-like structures surrounded by delicate fibrovascular stroma [5–7, 11]. Immunohistochemically tumor cells are positive for cytokeratin, estrogen receptors (ER), progesterone receptors (PR), neuron specific enolase (NSE), chromogranin A, and/or synaptophysin [4–7, 18, 21]. NECB are more likely to be ER/PR positive and HER-2 negative [6, 15, 22]. The intraductal components could be both inside and outside of the invasive area. Vascular permeation and lymphatic permeation are also described [3].

Neuroendocrine tumors are composed of endocrine cells that are normally found in nervous tissue and endocrine system all over the body. These tumors include pancreatic neoplasms, paraganglioma, carcinoid tumors, pheochromocytoma, medullary thyroid carcinoma, and small cell carcinoma [21]. Most common sites of involvement are lungs and gastrointestinal tract [19]. Primary neuroendocrine cancer of the breast must be distinguished from a metastatic lesion from other sites. Small cell type of NEBC, which is CK7 positive and CK20 negative, is morphologically similar to small cell lung carcinoma, negative for both markers [18].

Most NECB are of high histologic grade, grade III (G3), while invasive breast carcinoma, NOS, is usually grade II (G2) (*P* < 0.0001). Most NEBC are ER and PR positive. Neuroendocrine differentiation is an independent adverse prognostic factor for both disease-specific and overall survival (both *P* < 0.0001) [2]. When compared with invasive breast carcinoma, NOS, NECB is associated with shorter survival [2, 23, 24]. Some authors believe that the most important factor is tumor grade (G) and there is no difference in prognosis of NECB and other mammary carcinomas [25, 26]. Important features for good prognosis are early stage, absence of lymph-node metastases, and positive ER and PR status [5, 10, 26].

There is no established standard treatment protocol because so few cases of primary NECB have been described in the literature [21]. Treatment is similar to that for other conventional types of invasive breast carcinomas and prognosis varies [7]. Therapeutic interventions depend on the size of the tumor, location, and clinical stage [20, 24, 25]. The general consensus is to treat small cell NECB with chemotherapy regiments for small cell lung carcinoma [18, 19, 21].

In conclusion, primary neuroendocrine carcinoma of the breast is a rare tumor, classified as type of invasive mammary carcinoma with distinctive histopathological features. Prognosis of NECB is not different from that of other invasive breast carcinomas and the most important prognostic factor is tumor grade (G). There is no standard treatment and patients should be treated similarly to patients with invasive ductal carcinoma, NOS, whose choice of therapy depends on tumor's size, degree of differentiation, clinical stage, and hormonal status.
